# Green Extraction Strategies and Bioactivity of *Rheum cordatum* Losinsk: Antioxidant, Antimicrobial, and Molecular Docking Insights

**DOI:** 10.3390/plants14071071

**Published:** 2025-04-01

**Authors:** Madina Amangeldinova, Mehmet Ersatır, Adem Necip, Mehmet Cimentepe, Nataliya Kudrina, Nina Terletskaya, Ozge Oztürk Cimentepe, Oguz Cakır, Mustafa Abdullah Yilmaz, Metin Yildirim

**Affiliations:** 1 Faculty of Biology and Biotechnology, Al-Farabi Kazakh National University, Al-Farabi Avenue 71, Almaty 050040, Kazakhstan; madu.ma@mail.ru (M.A.); kudrina_nat@mail.ru (N.K.); teni02@mail.ru (N.T.); 2 Institute of Genetics and Physiology, Al-Farabi 93, Almaty 050040, Kazakhstan; 3 Department of Chemistry, Faculty of Art and Science, Cukurova Universiry, Adana 01330, Türkiye; mehmetersatir8@gmail.com; 4 Department of Pharmacy Services, Vocational School of Health Services, Harran University, Sanliurfa 63100, Türkiye; ademnecip@harran.edu.tr; 5 Department of Pharmaceutical Microbiology, Faculty of Pharmacy, Harran University, Sanliurfa 63100, Türkiye; mehmet.cimentepe@harran.edu.tr; 6 Department of Pharmacology, Faculty of Pharmacy, Harran University, Sanliurfa 63100, Türkiye; ozge.cimentepe@harran.edu.tr; 7 Department of Nutrition and Dietetics, Faculty of Health Sciences, Dicle University, Diyarbakir 21280, Türkiye; ocakir44@gmail.com; 8 Department of Analytical Chemistry, Faculty of Pharmacy, Dicle University, Diyarbakir 21280, Türkiye; mustafaabdullahyilmaz@gmail.com; 9 Department of Biochemistry, Faculty of Pharmacy, Harran University, Sanliurfa 63100, Türkiye

**Keywords:** *Rheum cordatum* Losinsk, ultrasound-assisted extraction, supercritical CO_2_ extraction, subcritical ethyl alcohol extraction, antioxidant, antimicrobial, molecular docking

## Abstract

This study aimed to compare the efficiency of different green extraction methods for obtaining bioactive compounds from the roots of *Rheum cordatum* Losinsk and to evaluate their antioxidant and antimicrobial properties. The presence of some important phytochemicals in the extracts obtained using ultrasound-assisted extraction (UAE), subcritical ethanol extraction (Sbc-EtOH), and supercritical CO_2_ (ScCO_2_) extraction was determined by LC-MS/MS, and their antioxidant and antimicrobial properties were examined against *Staphylococcus aureus*, *Enterococcus faecalis*, *Pseudomonas aeruginosa*, and *Escherichia coli*. The goal was to determine the optimal extraction conditions that maximize the yield of bioactive compounds while preserving their biological properties. Different pressures (100 bar and 400 bar) were tested in UAE extraction, different solvents and times were tested in Sbc-EtOH extraction, and different pressures were tested in ScCO_2_ extraction. Most of the 53 important phenolic compounds have been extracted using the ScCO_2_ extraction method, either exclusively or in the highest amounts. It has been observed that more and higher amounts of phenolic compounds were extracted at lower pressure. The highest antioxidant activity was exhibited by the ScCO_2_ extracts. Additionally, the ScCO_2_-100 extract obtained at 100 bar showed strong antimicrobial activity, with a minimum inhibitory concentration (MIC) ranging from 31.25 to 250 μg/mL. Gallic acid, epicatechin gallate, epigallocatechin gallate, and catechin were found in extracts. Additionally, molecular docking studies against the 1QWZ, 2ANQ, 3H77, and 6QXS proteins revealed that epicatechin exhibited docking scores of −6.127, −9.479, −5.836, and −7.067 kcal/mol, respectively.

## 1. Introduction

Plant extracts play a crucial role in modern medicine and cosmetology due to their numerous chemical substances, which can be used to prevent a wide range of disorders [1]. The study of plant extracts merges traditional knowledge of folk medicine with the advancements of modern science, not only preserving ethnobotanical knowledge but also identifying new bioactive compounds with potential pharmaceutical applications [2,3]. Additionally, the vast diversity of the plant kingdom provides significant chemical and biochemical variety.

The genus *Rheum* belongs to the family Polygonaceae and includes perennial plants commonly known as rhubarb. Rhubarb is among the oldest and most economically significant herbs [4]. Morphological characteristics typical of the genus Rheum include thick roots, hollow erect stems, and small inflorescences of white-green or purple-red flowers [5]. These plants are widely distributed in Asia, particularly in regions such as Tibet, Mongolia, China, and Siberia, with some species also introduced to Europe and North America [6]. There is substantial evidence of rhubarb use in China since the third millennium BCE as a laxative [7], as first described by the author of *The Divine Farmer’s Classic of Materia Medica*, Shen Nong. It has been used to treat conditions such as constipation, diabetic nephropathy, chronic renal failure, acute pancreatitis, and gastrointestinal bleeding. The genus Rheum is known for its properties in reducing heat, clearing fire, cooling the blood, and dispersing blood stasis [8]. In recent decades, extensive research on the genus Rheum has further demonstrated its antidiabetic [9], antioxidant [10,11,12], and antimicrobial properties [13,14,15].

Currently, it is known that all plants of the genus *Rheum* contain secondary metabolites such as anthraquinones [16], flavonoids [17], tannins [18], and stilbenoids [19] in varying concentrations. These biologically active compounds have already been applied in pharmaceuticals and are used in combination therapy for various pathological conditions. Biologically active substances typically exhibit lower toxicity and higher bioavailability. According to WHO data from 2023, plant-derived biologically active compounds now account for 40% of the global pharmaceutical industry [20]. Considering the principle of relatedness of species, it can be assumed that Kazakhstani representatives of the genus Rheum have the above properties. *R.cordatum*, which grows in Kazakhstan, is a promising yet understudied species. The study of these compounds in *Rheum cordatum* Losinsk is of significant importance in pharmacy due to their numerous biological properties. Effective extraction strategies for these compounds ensure their wide application in medicine [21]. Traditional extraction methods, including maceration, hydro distillation, and percolation, remain widespread. Such classical extractions of secondary metabolites have a number of disadvantages, such as the use of hazardous solvents, the length of the process, and the low efficiency of extraction of target compounds, which is fraught with the loss of a large amount of biologically active substances [22]. These shortcomings have led to the development of more modern extraction methods for plant materials. Modern and environmentally friendly extraction methods include supercritical CO_2_ extraction (SC-CO_2_), subcritical ethanol extraction (sbcEtOH-E), and ultrasound-assisted extraction (UAE), among others [23,24].

Supercritical extraction using carbon dioxide (Sc-CO_2_) is considered one of the methods derived from superfluid extraction (SFE) technology. It is widely used as a ‘green strategy’ for the efficient extraction of valuable chemical compounds from various plant materials [25,26]. Sc-CO_2_ enables extraction at low temperatures, which is crucial for preserving thermolabile bioactive compounds [27]. This advantage is particularly beneficial for extracting compounds such as carotenoids, flavonoids, and phenols from fruits and plants [28]. Its supercritical state, achieved at a temperature of 31 °C and a pressure of 72.9 bar, exhibits gas and liquid properties, making it ideal for extracting secondary metabolites from natural resources. To effectively extract bioactive substances from plant materials with minimal loss of their beneficial properties, optimal temperatures and pressures are required, which may vary depending on the plant composition. For instance, in studies conducted by Villacís-Chiriboga et al. (2021), the goal was to extract carotenoids from the peel and pulp of mango (*Mangifera indica* L.). The extraction was performed at a temperature of 55 °C and a pressure of 35 MPa, with the addition of 20% ethanol. As a result, the peel contained 4.1 times more bioactive compounds than the pulp [29].

Polar bioactive compounds were extracted from *Vernonia amygdalina* L. at a temperature of 60 °C and a pressure of 250 bar. These parameters resulted in the highest yield [30]. Therefore, it can be hypothesized that adjusting parameters such as pressure and temperature and adding co-solvents like ethanol can enhance the yield of specific compounds.

Ultrasound-assisted extraction (UAE) is another equally modern and advanced method for extracting bioactive compounds. This method enables the extraction of secondary metabolites with higher yields in a shorter time compared to traditional methods and minimizes the degradation of thermosensitive bioactive compounds (BC) [31]. In recent years, UAE has been actively used to extract biologically active components from various sources, such as *Allium ursinum* L. [32], *Cynara scolymus* L. [33], *Annona muricate* [34], and *Ribes nigrum* [35], among others. Moreover, ultrasound-assisted extraction is widely applied in the food industry as a proven green technology that effectively accelerates chemical reactions through cavitation [36]. The wave released during extraction causes structural changes in the plant matrix [37], promoting the release of biologically active chemical compounds from the plant matrix after cell disruption [38].

Subcritical extraction is also considered an environmentally friendly and modern method today, as it does not require additional energy sources such as microwave radiation or ultrasound [39]. This method encompasses several techniques, each distinguished by the solvents used and optimal conditions. For example, subcritical water extraction (SWE) employs water as the extractant at temperatures ranging from 100 °C to 374 °C [40], conducted at sufficiently high pressure to keep the water in a liquid state. Subcritical extraction is also carried out by adding ethanol, an eco-friendly method that ensures a high yield of compounds [41].

The aim of this study is to comparatively investigate the efficiency of different extraction methods for bioactive compounds from the roots of *Rheum cordatum* Losinsk using supercritical carbon dioxide extraction (Sc-CO_2_), ultrasound-assisted extraction (UAE), and subcritical ethanol extraction (sbcEtOH-E). The research focuses on identifying optimal conditions for maximizing the extraction of bioactive substances while preserving their biological properties. Previous studies, including our investigation of *Rheum tataricum* L. [3], have demonstrated that extraction efficiency and phytochemical composition are significantly influenced by the choice of extraction method. However, a direct comparison between *Rheum cordatum* Losinsk and other Rheum species in terms of bioactive compound composition and biological activities remains unexplored. This study addresses this gap by systematically evaluating the extraction efficiency, antioxidant potential, and antibacterial activity of *Rheum cordatum* Losinsk, providing critical insights into its pharmacological relevance. To the best of our knowledge, this is the first comprehensive study applying UAE, sbcEtOH-E, and Sc-CO_2_ to *Rheum cordatum* Losinsk, establishing a foundation for future phytopharmaceutical applications of this species.

## 2. Material and Methods

### 2.1. Plant Material

The subject of this research is the roots of *Rheum cordatum* Losinsk, collected on 20 April 2024, in the Korday district of the Zhambyl region of Kazakhstan, in the southern foothills of the Chu-Ili mountains (Collection Geolocation: 43°16′42.0″ N 74°51′23.0″ E, found on rocky slopes at approximately 1200 m above sea level). The collected plant was authenticated at the Institute of Botany and Phytointroduction of the Ministry of Education and Science of the Republic of Kazakhstan, herbarium identification number–*Rheum cordatum* Losinsk 0003459. The samples were dried in a vacuum at 45–50 °C and ground to a powder. The dried plant material was stored at Harran University in Şanlıurfa, Turkey.

### 2.2. Extraction Methods

#### 2.2.1. Ultrasound-Assisted Extraction (UAE)

Ultrasound-assisted extraction experiments were carried out using an Elmasonic Select 150 device. For each experiment, 2 g of *R. cordatum* roots and 30 mL of solvent were added to a 50 mL capped sample tube. The experiments were conducted at room temperature using two different solvents [methanol (MeOH) and ethanol (EtOH)] with two different extraction times (1 h and 4 h). The sample tubes were placed in the middle of the ultrasonic bath to ensure uniform exposure to ultrasound waves, optimizing cavitation effects and energy transfer. All extractions were performed in triplicate, and the extraction yield was calculated using Equation (1).

#### 2.2.2. Subcritical Ethanol Extraction (sbcEtOH-E)

The experiments were conducted using a device designed for subcritical water extraction. The subcritical ethanol extraction (sbcEtOH-E) was performed using 1 g of *Rheum cordatum* Losinsk roots at a temperature of 140 °C and pressures of 60 and 80 bar, with a 30 min static extraction time followed by a 20 min dynamic extraction using ethyl alcohol at a flow rate of 2 mL per minute. The extraction yield, expressed as the mass ratio of the extract to the starting dried material, was calculated using Equation (1). All extractions were conducted in triplicate.

[Y % = g of extract/g of dried material × 100](1)

#### 2.2.3. Supercritical Carbon Dioxide Extraction (ScCO_2_-E)

*Rheum cordatum* root extracts were prepared using a supercritical CO₂ extractor (Supercritical Extraction System SuperEx F series 500, MTA Teknoloji Ltd. Şti., Ankara, Türkiye). Briefly, 25 g of RC root (in a polyester pouch) was placed into the extractor vessel. The temperature values for the extractor, restrictor, and separator were set to 60 °C, 120 °C, and 60 °C, respectively. The pressure was set to 100 and 400 bar. Extraction yield was calculated by Equation (1) and the extractions were conducted in triplicate.

### 2.3. LC-MSMS Analysis

A previously developed and validated LC-MS/MS method was applied for the qualitative and quantitative evaluation of secondary metabolites, including phenolic compounds, flavonoids, and anthraquinones, in *Rheum cordatum* Losinsk roots [42]. Information regarding the validation procedures and chromatographic conditions of the method are provided in the Supplementary Material file.

### 2.4. Antioxidant Activity

#### 2.4.1. DPPH Radical Scavenging Activity Method

The DPPH free radical scavenging activity analysis was conducted using the DPPH (1,1-diphenyl-2-picrylhydrazyl) method (Blois, 1958) [43] with minor modifications as described in the study by Necip and Işık (2019). Plant extracts were prepared at a concentration of 1.00 mg/mL, and the final volume was adjusted to 2.0 mL. Subsequently, 0.5 mL of DPPH radical solution was added to the solutions, which were then vortexed and incubated in the dark for half an hour. Absorbance values were measured at a wavelength of 517 nm using a UV spectrophotometer. Trolox, BHA, and BHT were used as positive controls. The results were expressed as half-maximal inhibitory concentration (IC_50_, mg/mL) [44].

#### 2.4.2. ABTS Radical Scavenging Activity Method

The ABTS free radical scavenging activity analysis was performed with minor modifications based on the study by Necip et al. (2021) [45]. ABTS radicals were generated by adding 2.45 mM persulfate solution to 7 mM ABTS solution. ABTS radical (7 mM) was added to the prepared solutions, vortexed, and incubated in the dark for 30 min. Absorbance values were measured at 734 nm using a UV spectrophotometer. Standard antioxidants (Trolox, BHA, and BHT) were used as positive controls. The results were also expressed as IC_50_ (mg/mL).

#### 2.4.3. Cu^2+^-Cu^+^ Reducing Activity

The copper reducing power analysis was based on the method described by Apak et al. (2004) [46], which involves the stable complex formation between neocuproine and copper (I) and measuring the absorbance at 450 nm. The Cu^2+^-Cu^+^ reducing activity analysis was conducted with minor modifications based on the study by Necip et al. (2024) [47]. Plant extracts and Trolox at different concentrations were added to a test tube containing 0.25 mL neocuproine (7.5 mM), 0.25 mL NH_4_Ac (1 M), and 0.25 mL CuCl_2_ (0.01 M) in ethanol. Absorbance values were measured at a wavelength of 450 nm using a UV spectrophotometer. Water was used as a blank. The results were presented as mg TE/mL Trolox equivalent.

#### 2.4.4. Total Phenolic Content Determination

Phenolic component analysis was performed with several modifications based on the method used by Necip and Durgun (2022) [48]. For the experiment, 50 µL of standard plant extracts were taken, 1.00 mL of distilled water was added to each sample, followed by the addition of 25 µL Folin-Ciocalteu reagent, and the mixture was homogenized. After 3 min, 40 µL of 20% sodium carbonate (Na_2_CO_3_) was added to the prepared samples, vortexed, and incubated in the dark at room temperature for two hours. Absorbance values were measured at 760 nm using a UV spectrophotometer. The equivalent amount of gallic acid corresponding to the measured absorbance was calculated. The results were expressed as mg GAE/mg extract.

### 2.5. Antimicrobial Activity

#### 2.5.1. Test Organism and Growth Conditions

Four reference bacterial strains, namely *Staphylococcus aureus* ATCC 29213, *Enterococcus faecalis* ATCC 29212, *Escherichia coli* ATCC 35150, and *Pseudomonas aeruginosa* ATCC 27853, were included in this study. Cultures were prepared from stock cultures employing the streaking technique on blood agar (Oxoid, England) plates for bacteria. After overnight incubation, a single colony was used to inoculate the sterile broths: Mueller–Hinton broth (MHB) (Oxoid, England) for bacteria. The inoculated broths were then incubated overnight. The microbial cultures were diluted in MHB and adjusted to a 0.5 McFarland standard turbidity level (10^8^ CFU/mL) [49].

#### 2.5.2. Determination of Minimum Inhibitory Concentration (MIC)

The broth microdilution method was used to determine the MIC according to the National Committee for Clinical Laboratory Standards. The MIC value was determined for the extracts. The MIC value was detected by the broth microdilution method using 96- well microtiter plates. Two-fold serial dilutions of extracts were prepared by dilution with MHB to achieve a decreasing concentration range from 2000 to 3.91 µg/mL. The bacterial inoculates were prepared using overnight cultures, and suspensions were adjusted to 0.5 McFarland standard turbidity. Each well was inoculated with 10 μL of the bacterial suspension at a density of 10^5^ CFU/mL The plates were incubated for 24 h at 37 °C, for bacterial cultures. Microdilution plates were incubated for 24 h at 37 °C. Minimum inhibitory concentration (MIC) was determined visually and using a microplate spectrophotometer (BioTek Inc., Winooski, VT, USA) at 570 nm optical density (OD). The lowest concentration of extracts that demonstrated no growth was evaluated as the MIC. All studies were performed in triplicate.

The minimum bactericidal concentration (MBC) was determined by subculturing the 10 μL of broths used for the MIC assay onto Mueller–Hinton agar (Merck Germany, Darmstadt, Germany). The MBC was considered to be the lowest concentration of extracts that resulted in the killing of 99.9% of the bacteria after incubation at 37 °C for 24 h.

### 2.6. Molecular Docking Studies

Molecular docking studies were described in detail in our earlier research. Briefly, the three-dimensional structures of the proteins were obtained from the https://www.rcsb.org/ database. Molecular docking analyses were conducted on proteins that are critical for bacteria targeted by the most common phytochemicals found in plant extracts and known for their antibacterial activity. The proteins used in these studies included 1QWZ, 2ANQ, 3H77, and 6QXS [50,51,52].

### 2.7. Statistical Analysis

All statistical analyses were performed with GraphPad Prism 9 (GraphPad Software Inc., Boston, MA, USA) using one-way ANOVA (Tukey’s multiple comparison test).

## 3. Results and Discussion

The roots of *Rheum cordatum* Losinsk were dried and extracted using different methods (ScCO_2_, sbcEtOH-E, and UAE) with two different solvents (EtOH and MeOH). Variations in these methods, as well as changes in solvent type, pressure, and extraction time, influenced the variety and ratio of active compounds obtained in the extracts.

The presence and quantity of 53 phytochemicals in the extracts obtained from plant roots under different extraction conditions were investigated using the LC-MS/MS method. The obtained results are presented in Table 1.

According to Table 1, 15 out of 53 important phytochemicals were detected in the extracts obtained from the roots of *Rheum cordatum* Losinsk, including quinic acid, gallic acid, protocatechuic acid, tannic acid, epigallocatechin gallate, caffeic acid, epicatechin gallate, p-coumaric acid, rutin, isoquercitrin, hesperidin, ellagic acid, quercetin, naringenin, and luteolin. Each of these phenolic compounds plays a crucial role in antioxidant, anti-inflammatory, antimicrobial, and cardioprotective activities, making them highly valuable for pharmaceutical and nutraceutical applications.

The concentration of these bioactive compounds varies significantly depending on the extraction method. Notably, epicatechin gallate was found in the highest concentration (83.53 mg/g) in UAE-EtOH-1h extracts, highlighting the efficiency of ultrasound-assisted extraction for flavan-3-ols. Epicatechin gallate and epigallocatechin gallate are potent free radical scavengers and antimicrobial agents [53]. Similarly, gallic acid, a well-known antioxidant and antimicrobial agent [54], was most abundant (76.97 mg/g) in ScCO_2_-100 bar extracts, suggesting that lower pressure enhances the recovery of this phenolic acid.

The ScCO_2_ extraction method proved to be the most effective for isolating a wide range of phytochemicals, with rutin, hesperidin, naringenin, and luteolin being highly concentrated in these extracts. These phytochemicals are known to reduce oxidative stress and inflammation [55,56]. Quinic acid and tannic acid, known for their hepatoprotective and astringent properties [57,58], contributing to their potential use in wound healing and gastrointestinal disorders, were predominantly found in Sbc-EtOH extracts, suggesting that subcritical ethanol extraction facilitates the extraction of these water-soluble phenolic acids. In contrast, epigallocatechin gallate and ellagic acid were best extracted using ultrasound-assisted extraction, further demonstrating the impact of cavitation on polyphenolic compound release. Ellagic acid has demonstrated anticancer potential [59], while quinic acid and p-coumaric acid play roles in metabolic regulation and hepatoprotection [60].

Several important compounds, such as fumaric acid, aconitic acid, vanillin, daidzin, coumarin, genistin, and amentoflavone, were not detected in any of the extracts, suggesting that their presence in *R. cordatum* may be negligible or that alternative extraction techniques are required for their isolation. Interestingly, 4-OH benzoic acid was detected exclusively in ScCO_2_-100 bar extracts, while salicylic acid was found in ScCO_2_-400 bar extracts, indicating that higher pressure may selectively enhance the extraction of certain phenolic acids.

Certain phytochemicals were only found in extracts from specific methods, further reinforcing the importance of method selection in phytochemical research. For example, chlorogenic acid was exclusively present in Sbc-EtOH extracts, whereas astragalin, nicotiflorin, hesperetin, apigenin, chrysin, and acacetin were detected only in ScCO_2_ extracts, emphasizing the selectivity of supercritical fluid extraction for flavonoids. Conversely, cyranoside was completely absent from ScCO_2_ extracts, which may be attributed to its higher solubility in polar solvents.

Overall, these findings underscore the critical role of extraction parameters in maximizing the yield of bioactive compounds. The results demonstrate that ScCO_2_ extraction at 100 bar offers a highly efficient and environmentally friendly strategy for obtaining phenolic acids and flavonoids from R. cordatum. Meanwhile, ultrasound-assisted extraction excels at recovering catechins, and subcritical ethanol extraction is beneficial for water-soluble compounds. The observed distribution of phytochemicals highlights the pharmacological potential of *R. cordatum*, supporting its application in the development of antioxidant, antimicrobial, and anti-inflammatory agents.

### 3.1. Antioxidant Activity

The antioxidant activity of *Rheum cordatum* Losinsk root extracts was evaluated using three assays for DPPH radical scavenging activity, ABTS radical scavenging activity, and Cuprac reducing power. Additionally, the total phenolic content (TPC) was quantified. The results of these analyses, including the TPC values and the antioxidant activity determined by DPPH, ABTS, and Cuprac assays, are summarized in Table 2.

DPPH Radical Scavenging Activity

The ability of the extracts to neutralize DPPH free radicals was assessed based on IC_50_ values (mg/mL), where lower values indicate higher antioxidant activity. The best results were obtained with supercritical CO_2_ extraction at 100 bar (ScCO_2_-100), which exhibited the lowest IC_50_ (0.0348 mg/mL), indicating the highest antioxidant potential. A similar result was observed for ScCO_2_-400 (IC_50_ = 0.0352 mg/mL), confirming the efficiency of supercritical extraction.

Ultrasound-assisted extraction (UAE) demonstrated moderate DPPH activity, with UAE-MeOH-1h (0.0606 mg/mL) and UAE-EtOH-1h (0.0645 mg/mL) exhibiting the best performance. The lowest radical scavenging capacity was observed for subcritical ethanol extraction (sbcEtOH), where IC_50_ values ranged from 0.1122 mg/mL (sbcEtOH-140-60) to 0.1297 mg/mL (sbcEtOH-140-80), indicating the weakest antioxidant activity among all methods.

A study by Raudsepp et al. (2008) reported that the DPPH radical scavenging activity of ethanol extracts from *R. rhaponticum* L. ranged from 25% to 77%. In comparison, our results for *R. cordatum* extracts show a DPPH scavenging range of 60% to 75%, which is consistent with these findings. However, our study provides quantitative IC_50_ values, demonstrating that the ScCO_2_ method enhances antioxidant activity [61].

Similarly, Zhumashova et al. (2019) reported that ethanol and water extracts of R. cordatum roots and stems showed DPPH radical scavenging activity of 83.7% and 92.7%, respectively. Our study presents more detailed results, including IC_50_ values for different extraction methods based on DPPH analysis. The extraction method using supercritical CO_2_ (100 atm) exhibited the highest antioxidant activity, with an IC_50_ value of 0.0348 mg/mL. Both studies confirm the strong antioxidant potential of *Rheum cordatum*. However, our research emphasizes the effectiveness of modern extraction methods, such as ScCO_2_, whereas Zhumashova et al. employed traditional extraction methods (ethanol, water) [11].

A comparison with *Rheum tataricum (R. tataricum* L.) extracts (our previous study) shows that the DPPH radical scavenging activity of *R. tataricum* extracts ranged between 0.0173 mg/mL and 0.0400 mg/mL [3]. The sbcEtOH-140-80 and sbcEtOH-140-60 extracts of R. tataricum exhibited the strongest DPPH scavenging effects (IC_50_ = 0.0173 mg/mL and 0.0182 mg/mL), indicating a higher antioxidant potential than *R. cordatum*. This suggests that subcritical ethanol extraction is more effective for *R. tataricum*, while supercritical CO_2_ extraction is optimal for *R. cordatum*.

In comparison with *Rheum khorasanicum* (Amirkhosvari et al., 2023), the antioxidant activity of *R.khorasanicum* was investigated using DPPH and FRAP assays [62]. The April-collected extract showed IC_50_ value od 0.038 mg/mL, comparable to our *R. cordatum* extracts (IC_50_: 0.0348 mg/mL). These findings confirm that both species exhibit significant antioxidant activity.

ABTS Radical Scavenging Activity

The antioxidant capacity against ABTS radicals was highest in ultrasound-assisted extracts (UAE-MeOH-1h and UAE-EtOH-1h), which exhibited the lowest IC_50_ values of 0.0056 mg/mL and 0.0055 mg/mL, respectively, surpassing all other methods. Supercritical CO_2_ extraction also demonstrated strong ABTS activity, with ScCO_2_-100 (0.0273 mg/mL) and ScCO_2_-400 (0.0256 mg/mL) yielding favorable results. In contrast, subcritical extraction exhibited the weakest performance, with IC_50_ values ranging from 0.0078 to 0.0080 mg/mL. These findings highlight the high efficiency of ultrasound-assisted extraction (UAE) in ABTS radical scavenging, particularly when ethanol is used as a solvent.

For *R. tataricum*, the ABTS radical scavenging activity was higher, with IC_50_ values ranging from 0.0027 mg/mL to 0.0275 mg/mL, indicating that UAE and subcritical ethanol extraction were higher in *R. tataricum* compared to *R. cordatum* [3].

Cuprac Assay

In terms of the Cu^2+^-Cu^+^ reduction activity, the Trolox equivalents of the extracts ranged from 0.0081 to 0.0236 mg TE/mL. The highest values were found in UAE-MeOH-1h and UAE-EtOH-1h The total phenolic content in the extracts was calculated as gallic acid equivalents. The lowest total phenolic content was found in the ScCO_2_ 100-60 extract (178.245 mg GAE/mL), and the highest was found in UAE-EtOH-1h (208.066 mg GAE/mL).

For *R. tataricum*, the Cuprac values ranged from 0.0058 mg TE/mL to 0.0138 mg TE/mL, with the highest values found in UAE-E-2h and UAE-M-2h. This suggests that ultrasound-assisted extraction enhances the reducing power of both *R. cordatum* and *R. tataricum*, but *R. cordatum* exhibits slightly higher reducing activity [3].

Total Phenolic Content (TPC)

The phenolic content of the extracts serves as an essential indicator of their antioxidant properties. Ultrasound-assisted extraction (UAE) resulted in the highest phenolic content, particularly in UAE-EtOH-1h (208.066 mg GAE/g) and UAE-MeOH-1h (195.355 mg GAE/g), confirming the efficiency of UAE in preserving phenolic compounds. Supercritical CO_2_ extraction yielded moderate TPC values (ScCO_2_-100 = 178.245 mg GAE/g, ScCO_2_-400 = 188.022 mg GAE/g), while subcritical ethanol extraction resulted in the lowest TPC values (sbcEtOH-140-60 = 176.289 mg GAE/g, sbcEtOH-140-80 = 189.002 mg GAE/g).

For *R. tataricum*, the total phenolic content ranged from 182.64 mg GAE/mL to 213.44 mg GAE/mL, with UAE-M-4h showing the highest TPC. This indicates that *R. tataricum* generally has a higher phenolic content than *R. cordatum*, particularly when extracted using UAE methods. Phenolic compounds are typically found in foods such as fruits, vegetables, and grains, and the antioxidant capacities of these foods are directly linked to their phenolic content. The findings of this study also indicate that *Rheum cordatum* roots contains phenolic compounds that provide antioxidant effects.

### 3.2. In Vitro Antimicrobial Activity

The bacterial strains *S. aureus*, *E. faecalis*, *P. aeruginosa*, and *E. coli* were utilized for the comparison of various parameters and extraction methods. According to the obtained data, among all extracts, sbcEtOH-E 140-60 exhibited the best antibacterial activity. Additionally, the green method, ScCO_2_ 100-60, showed strong antibacterial effects compared to other extracts. This extract, which also demonstrated the highest DPPH activity, exhibited significant antibacterial activity, with MIC values ranging from 31.25 to 250. The extracts ScCO_2_ 100-60 and ScCO_2_ 400-60 showed varying antibacterial activity due to the changes in the pressure parameters during extraction, which altered the extract content. The obtained extracts exhibited the highest antibacterial activity against the *E. faecalis* strain (MIC range 31.25 to 1000). When examining the effect of solvent variation on antibacterial activity in extracts obtained using the ultrasonic method, it was observed that the antibacterial activities of the UAE-EtOH-1h and UAE-MeOH-1h extracts were identical. Additionally, it was determined that obtaining extracts from different durations of UAE-MeOH did not result in any variation in antibacterial effect (Table 3).

For instance, in the study by Malik et al. (2018), *Bacillus megaterium* and *Pseudomonas aeruginosa* were used to evaluate the antibacterial activity of the methanolic extract of *Rheum emodi* Wall. The most pronounced activity was observed against *B. megaterium* (36.7 mm) and *P. aeruginosa* (39.6 mm) [63]. In the work by Rolta et al. (2020), a chloroform subfraction of *Rheum emodi* Wall. was tested against *S. aureus*, *E. coli*, and *K. pneumoniae*, where the strongest activity was shown against *E. coli*, with MIC of 3.91 µg/mL [64]. In the study by Canli et al. (2016), the ethanolic extract of *Rheum rhabarbarum* was tested on a broad spectrum of microorganisms, including *Bacillus subtilis*, *Candida albicans*, *Enterobacter aerogenes*, *Enterococcus durans*, *Enterococcus faecalis*, *Enterococcus faecium*, *Escherichia coli*, *Klebsiella pneumoniae*, *Listeria monocytogenes*, *Pseudomonas fluorescens*, and *Staphylococcus aureus* [65]. The most significant activity was observed against *E. faecium*, with inhibition zones of 25 mm at 50 µL and 28 mm at 100 µL of extract. In the study by Onem et al. (2020), methanolic, methanol-chloroform, and aqueous extracts of *Rheum ribes* L. were tested against *S. aureus*, methicillin-resistant *S. aureus* (MRSA), *Bacillus cereus*, *Enterococcus faecalis*, and *Listeria monocytogenes*. The most notable results were obtained with the methanolic extract against MRSA (19.7 mm) and *B. cereus* (19.7 mm) [66].

The antibacterial activity of *Rheum cordatum* Losinsk extracts was compared with that of *Rheum tataricum* L, previously investigated using similar extraction techniques [3]. The minimum inhibitory concentration (MIC) values for both species ranged between 31.25 and 250 μg/mL, with *Enterococcus faecalis* being the most susceptible bacterium (MIC = 31.25 μg/mL for both species). In both studies, ScCO_2_ and sbcEtOH-E extractions demonstrated the highest antibacterial potential, indicating that these green extraction methods effectively concentrate bioactive compounds with antimicrobial properties. However, a key difference was observed in the MIC values for *Pseudomonas aeruginosa*, where *Rheum tataricum* L extracts exhibited lower MIC values (125–500 μg/mL) compared to *Rheum cordatum* Losinsk (125–1000 μg/mL), suggesting a stronger effect against this Gram-negative bacterium. A notable phytochemical difference between the two species was the presence of epigallocatechin gallate in *Rheum cordatum* Losinsk, which was absent in *Rheum tataricum* L. This compound has been associated with enhanced antibacterial activity, particularly against Gram-negative bacteria. Both species, however, were rich in gallic acid, catechin, epicatechin gallate, protocatechuic acid, and chlorogenic acid, which are known to contribute to antimicrobial effects.

The study of the antimicrobial activity of the extract from *Rheum cordatum* Losinsk established the MIC for *Staphylococcus aureus* and *Escherichia coli* at 250 µg/mL, indicating moderate antibacterial activity compared to other Rheum species such as *Rheum emodi* and *Rheum rhabarbarum*. At the same time, the extract from *Rheum cordatum* demonstrated significant activity against *Enterococcus faecalis*, with an MIC of 31.25 µg/mL, which exceeds the values reported in previous studies for other Rheum species. This indicates the high antimicrobial potential of *Rheum cordatum* root extract against *E. faecalis* (MIC range 31.25 to 1000 µg/mL), especially when using advanced extraction methods such as supercritical CO_2_ extraction.

### 3.3. Molecular Docking Studies

According to the LC-MS/MS analysis of the extracts, the most common compounds identified were epicatechin, epicatechin gallate, epigallocatechin gallate, and gallic acid. The antibacterial properties exhibited by these extracts are likely attributable to these compounds. Therefore, molecular docking studies were conducted to assess the interactions of these compounds with bacterial proteins: 1QWZ (*S. aureus*), 2ANQ (*E. coli*), 3H77 (*P. aeruginosa*), and 6QXS (*E. faecalis*).

**Figure 1:**
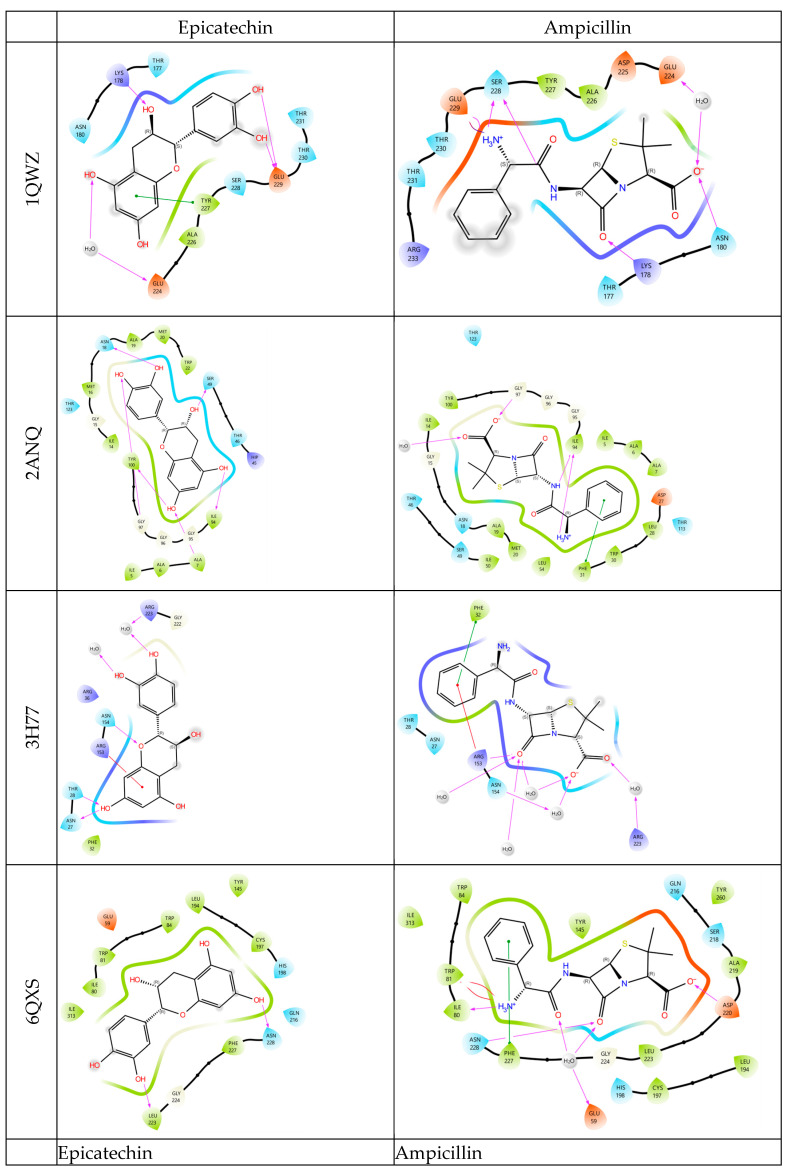

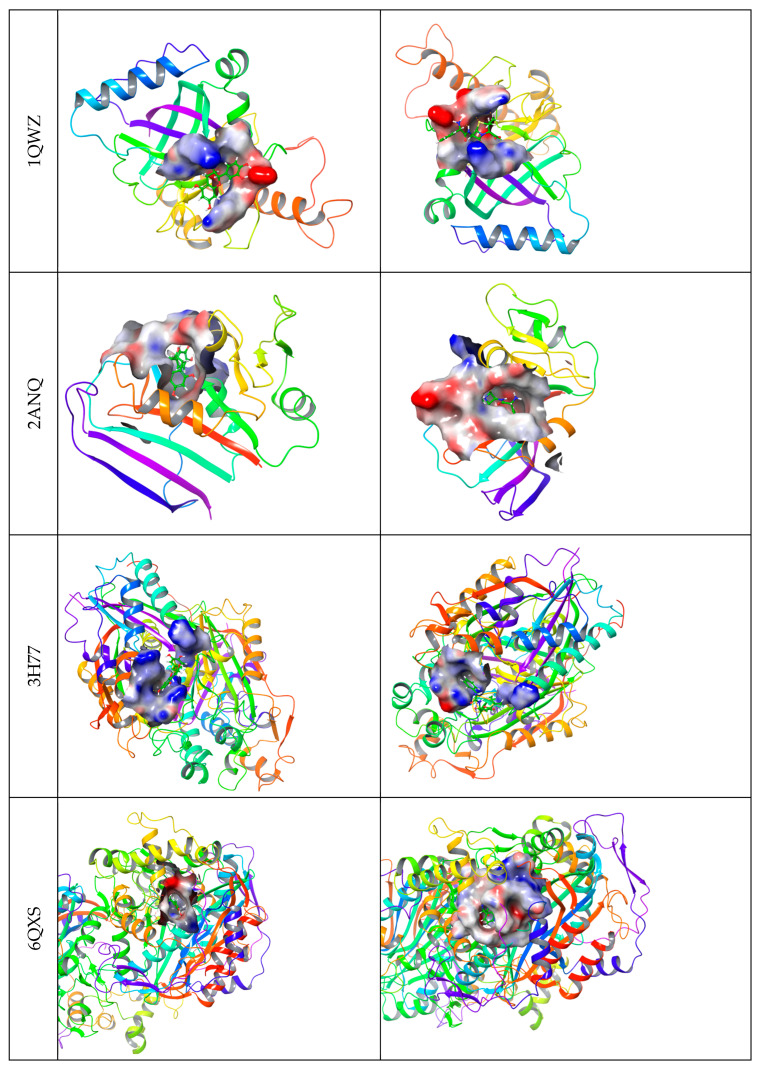
2D and 3D binding interactions of epicatechin, epicatechin gallate, epigallocatechin gallate, gallic acid, and ampicillin in the active site with the *S. aureus* PDB ID: (1QWZ), *E. coli* (PDB ID: 2ANQ), *P. aeruginosa* (PDB ID: 3H77), and *E. faecalis* (PDB ID: 6QXS) receptor.

For the 1QWZ protein, the docking scores ranged from −6.127 to −3.410 kcal/mol, with epicatechin showing the highest binding affinity, surpassing the efficacy of the positive control, ampicillin. In the case of the 2ANQ protein, epicatechin and epigallocatechin gallate exhibited docking scores of −9.479 and −9.385 kcal/mol, respectively, both demonstrating higher binding affinities than the positive control. However, for the 3H77 and 6QXS proteins, all tested molecules showed lower binding affinities compared to ampicillin (Table 4). Epicatechin formed hydrogen bonds with the 1QWZ protein through LYS178, GLU224, and GLU229 residues. In the 2ANQ protein, hydrogen bonding interactions were observed with ASN18, TYR100, SER49, and ILE94 residues. For the 3H77 protein, hydrogen bonds were established through ASN154, THR28, and ASN27 residues. In the case of the 6QXS protein, hydrogen bonding interactions occurred via LEU223 and ASN228 residues (Figure 1).

## 4. Conclusions

Based on the obtained data, the most effective extract was determined to be the one extracted with ScCO_2_-100, a fast and environmentally friendly method. A significant portion of the 53 important phenolic compounds was either only observed in ScCO_2_ extracts or extracted in maximum amounts. In this context, ScCO_2_ extraction has emerged as a valuable extraction method for obtaining important phytochemicals from the roots of *Rheum cordatum* Losinsk. Comparison with other Rheum species revealed that *Rheum cordatum* Losinsk exhibits comparable or superior antibacterial activity, particularly against Enterococcus faecalis (MIC = 31.25 μg/mL, which was lower than MIC values reported for *Rheum emodi* Wall and *Rheum rhabarbarum*) However, against *Pseudomonas aeruginosa*, extracts from *Rheum tataricum* L. demonstrated lower MIC values (125–500 μg/mL vs. 125–1000 μg/mL for *Rheum cordatum* Losinsk), suggesting species-dependent antibacterial properties.

The antioxidant activity of *Rheum cordatum* Losinsk was found to be within the range of standard antioxidants such as ascorbic acid and gallic acid, demonstrating strong radical scavenging potential. A key finding was the presence of epigallocatechin gallate, a compound absent in *Rheum tataricum* L., which may contribute to the distinct bioactivity of *Rheum cordatum* Losinsk. Variations in temperature, pressure, and solvent composition significantly influenced the phytochemical profile, supporting the importance of method selection in optimizing biological activity.

Molecular docking studies revealed strong interactions of epicatechin with bacterial and antioxidant-related protein targets (1QWZ, 2ANQ, 3H77, and 6QXS), with docking scores of −6.127, −9.479, −5.836, and −7.067 kcal/mol, respectively, supporting its potential role in the observed bioactivity. These findings provide a molecular basis for the antioxidant and antibacterial effects of the identified compounds.

This study represents the first comprehensive analysis of *Rheum cordatum* Losinsk using multiple extraction techniques, providing critical insights into the relationship between extraction conditions, phytochemical composition, and biological activity. Future research should focus on lower-pressure ScCO_2_ extractions to selectively enrich different phenolic compounds and further in vivo evaluations to validate their pharmacological potential. These findings contribute to the development of optimized extraction strategies for high-yield bioactive compounds, supporting their potential application in pharmaceutical and therapeutic formulations.

## Figures and Tables

**Table 1:** The phytochemicals and their quantities in the different extracts of *Rheum cordatum* Losinsk roots.

No.	Analytes	ScCO2-100	ScCO2-400	SbcEtOH-60	SbcEtOH-80_	UAE-EtOH-1h	UAE-EtOH-4h	UAE-MeOH-1h	UAE-MeOH-4h
1	Quinic acid	0.421	0.297	1.616	2.357	0.373	0.352	0.644	0.503
2	Fumaric aid	N.D.	N.D.	N.D.	N.D.	N.D.	N.D.	N.D.	N.D.
3	Aconitic acid	N.D.	N.D.	N.D.	N.D.	N.D.	N.D.	N.D.	N.D.
4	Gallic acid	76.967	42.596	54.526	72.364	49.735	44.883	46.59	48.396
5	Epigallocatechin	N.D.	N.D.	N.D.	N.D.	N.D.	N.D.	N.D.	N.D.
6	Protocatechuic acid	3.29	1.613	1.602	2.836	0.847	0.682	0.9	0.968
7	Catechin	3.806	N.D.	5.112	6.132	6.989	4.621	6.208	6.37
8	Gentisic acid	N.D.	N.D.	N.D.	N.D.	N.D.	N.D.	N.D.	N.D.
9	Chlorogenic acid	N.D.	N.D.	0.133	0.681	N.D.	N.D.	N.D.	N.D.
10	Protocatechuic aldehyde	0.263	0.584	0.369	0.502	N.D.	0.035	0.068	0.064
11	Tannic acid	3.661	1.738	2.895	5.97	1.263	1.693	2.573	3.592
12	Epigallocatechin gallate	18.167	2.729	21.469	14.835	30.557	26.634	29.218	28.09
13	Cynarin	N.D.	N.D.	N.D.	N.D.	N.D.	N.D.	N.D.	N.D.
14	4-OH Benzoic acid	1.693	1.37	N.D.	0.463	N.D.	N.D.	N.D.	N.D.
15	Epicatechin	8.143	N.D.	20.751	20.337	24.413	15.652	20.086	19.913
16	Vanilic acid	N.D.	N.D.	N.D.	N.D.	N.D.	N.D.	N.D.	N.D.
17	Caffeic acid	0.543	0.165	0.074	0.145	0.052	0.039	0.043	0.038
18	Syringic acid	N.D.	N.D.	N.D.	N.D.	N.D.	N.D.	N.D.	N.D.
19	Vanillin	N.D.	N.D.	N.D.	N.D.	N.D.	N.D.	N.D.	N.D.
20	Syringic aldehyde	N.D.	N.D.	N.D.	N.D.	N.D.	N.D.	N.D.	N.D.
21	Daidzin	N.D.	N.D.	N.D.	N.D.	N.D.	N.D.	N.D.	N.D.
22	Epicatechin gallate	77.341	9.998	61.431	50.102	83.53	73.078	80.27	79.744
23	Piceid	N.D.	N.D.	N.D.	N.D.	N.D.	N.D.	N.D.	N.D.
24	p-Coumaric acid	0.855	0.491	0.093	0.147	0.08	0.086	0.12	0.065
25	Ferulic acid-D3-ISh	N.A.	N.A.	N.A.	N.A.	N.A.	N.A.	N.A.	N.A.
26	Ferulic acid	N.D.	N.D.	N.D.	N.D.	N.D.	N.D.	N.D.	N.D.
27	Sinapic acid	N.D.	N.D.	N.D.	N.D.	N.D.	N.D.	N.D.	N.D.
28	Coumarin	N.D.	N.D.	N.D.	N.D.	N.D.	N.D.	N.D.	N.D.
29	Salicylic acid	0.027	0.035	0.018	N.D.	N.D.	N.D.	N.D.	N.D.
30	Cyranoside	N.D.	N.D.	0.05	0.041	0.048	0.043	0.054	0.046
31	Miquelianin	N.D.	N.D.	N.D.	N.D.	N.D.	N.D.	N.D.	N.D.
32	Rutin-D3-IS	N.A.	N.A.	N.A.	N.A.	N.A.	N.A.	N.A.	N.A.
33	Rutin	4.778	1.383	0.24	0.214	0.32	0.241	0.247	0.211
34	isoquercitrin	1.865	0.209	0.346	0.318	0.452	0.398	0.407	0.368
35	Hesperidin	3.791	1.029	0.197	0.716	0.284	0.182	0.254	0.212
36	o-Coumaric acid	N.D.	N.D.	N.D.	N.D.	N.D.	N.D.	N.D.	N.D.
37	Genistin	N.D.	N.D.	N.D.	N.D.	N.D.	N.D.	N.D.	N.D.
38	Rosmarinic acid	N.D.	N.D.	N.D.	N.D.	N.D.	N.D.	N.D.	N.D.
39	Ellagic acid	1.671	0.545	0.842	0.513	3.425	2.501	3.254	2.507
40	Cosmosiin	N.D.	N.D.	N.D.	N.D.	N.D.	N.D.	N.D.	N.D.
41	Quercitrin	0.204	N.D.	0.058	0.037	0.073	0.069	0.065	0.058
42	Astragalin	0.166	N.D.	N.D.	N.D.	N.D.	N.D.	N.D.	N.D.
43	Nicotiflorin	1.069	0.221	N.D.	N.D.	N.D.	N.D.	N.D.	N.D.
44	Fisetin	N.D.	N.D.	N.D.	N.D.	N.D.	N.D.	N.D.	N.D.
45	Daidzein	N.D.	N.D.	N.D.	N.D.	N.D.	N.D.	N.D.	N.D.
46	Quercetin-D3-IS	N.A.	N.A.	N.A.	N.A.	N.A.	N.A.	N.A.	N.A.
47	Quercetin	0.837	0.09	0.093	0.074	0.075	0.08	0.115	0.056
48	Naringenin	0.114	0.053	0.013	0.018	0.015	0.013	0.013	0.016
49	Hesperetin	0.058	N.D.	N.D.	N.D.	N.D.	N.D.	N.D.	N.D.
50	Luteolin	0.016	0.004	0.003	0.003	0.003	0.003	0.003	0.004
51	Genistein	N.D.	N.D.	N.D.	N.D.	N.D.	N.D.	N.D.	N.D.
52	Kaempferol	0.116	0.035	0.009	0.012	0.015	N.D.	0.012	0.01
53	Apigenin	0.016	0.011	N.D.	N.D.	N.D.	N.D.	N.D.	N.D.
54	Amentoflavone	N.D.	N.D.	N.D.	N.D.	N.D.	N.D.	N.D.	N.D.
55	Chrysin	0.09	0.075	N.D.	N.D.	N.D.	N.D.	N.D.	N.D.
56	Acacetin	0.012	0.023	N.D.	N.D.	N.D.	N.D.	N.D.	N.D.

N.D.: Not detected, N.A.: Not applicable.

**Table 2:** Antioxidant activity of *Rheum cordatum* Losinsk extracts.

	DPPH (IC_50_ mg/mL)	R^2^	ABTS (IC_50_ mg/mL)	R^2^	CUPRAC (mg TE/mL)	Toplam Fenolik (mg GAE/g)
BHA	0.0023 ± 0.0002	0.994	0.0021 ± 0.0002	0.981		
BHT	0.0038	0.961	0.0034	0.993		
Trolox	0.0076	0.991	0.0041	0.998		
UAE-MeOH-1h	0.0606 ± 0.003 ^a^	0.964	0.0056 ± 0.0003 ^a^	0.994	0.0236 ± 0.0012	195.355 ± 9.75
UAE-EtOH-1h	0.0645 ± 0.003 ^a^	0.965	0.0055 ± 0.0003	0.995	0.0228 ± 0.0012	208.066 ± 10.4
UAE-MeOH-4h	0.0654 ± 0.003 ^a^	0.968	0.0058 ± 0.0003 ^a^	0.994	0.0195 ± 0.001 ^b,c^	204.644 ± 11
UAE-EtOH-4h	0.0659 ± 0.004 ^a^	0.969	0.0057 ± 0.0003 ^a^	0.995	0.0197 ± 0.001 ^b,c,d^	207.088 ± 11 ^c^
sbcEtOH-E 140-60	0.1122 ± 0.005 ^a,b,c,d,e^	0.964	0.0080 ± 0.0004 ^a,b,c,d,e^	0.964	0.0113 ± 0.001 ^b,c,d,e^	176.289 ± 9 ^b^
sbcEtOH-E 140-80	0.1297 ± 0.006 ^a,d,e,f^	0.966	0.0078 ± 0.0003 ^a,c,d,e^	0.965	0.0105 ± 0.001 ^b,c,d,e^	189.002 ± 10 ^c,e^
ScCO_2_ 100-60	0.0352 ± 0.002 ^a,b,c,d,e^	0.997	0.0273 ± 0.0013	0.995	0.0139 ± 0.001 ^b,c,d,e,g^	178.245 ± 9 ^b,e,f^
ScCO_2_ 400-60	0.0348 ± 0.002 ^a,b,c,d,e^	0.992	0.0256 ± 0.0012	0.992	0.0081 ± 0.0006 ^b,c,d,e,f,h^	188.022 ± 10 ^c,e^

^a^ *p* < 0.05 compared to BHA, ^b^ *p* < 0.05 compared to UAE-MeOH-1h, ^c^ *p* < 0.05 compared to UAE-EtOH-1h, ^d^ *p* < 0.05 compared to UAE-MeOH-4h, ^e^ *p* < 0.05 compared to UAE-EtOH-4h, ^f^ *p* < 0.05 compared to sbcEtOH-E 140-60, ^g^ *p* < 0.05 compared to sbcEtOH-E 140-80, ^h^ *p* < 0.05 compared to ScCO2 100-60.

**Table 3:** MIC (μg/mL) and bactericidal concentration (MBC; μg/mL) of extracts against Gram-positive and negative pathogenic bacteria.

	*S. aureus*		*E. faecalis*		*P. aeruginosa*		*E. coli*	
	MIC	MBC	MIC	MBC	MIC	MBC	MIC	MBC
UAE-EtOH-1h	250	250	125	125	250	500	500	500
UAE-EtOH-4h	500	1000	1000	2000	500	1000	500	1000
UAE-MeOH-1h	250	250	125	125	250	500	500	500
UAE-MeOH-4h	250	250	125	125	250	500	500	500
sbcEtOH-E 140-60	250	250	62.5	125	500	500	250	500
sbcEtOH-E 140-80	125	250	62.5	125	250	500	250	500
ScCO_2_ 100-60	250	500	31.25	250	250	250	250	500
ScCO_2_ 400-60	500	1000	62.5	500	500	500	500	1000
Ampicillin	*		*		31.25		3.9	

* Effective at all concentrations.

**Table 4:** Molecular docking scores and binding modes of compounds with the 1QWZ, 2ANQ, 3H77, and 6QXS receptor (kcal/mol).

	1QWZ		2ANQ		3H77		6QXS	
	Docking Score	Glide Emodel	Docking Score	Glide Emodel	Docking Score	Glide Emodel	Docking Score	Glide Emodel
Epicatechin	−6.127	−49.236	−9.479	−66.637	−5.836	−39.315	−7.067	−63.494
Epicatechin gallate	−3.410	−39.416	−6.769	−76.817	−4.955	−40.342	−7.616	−78.489
Epigallocatechin gallate	−3.915	−43.334	−9.385	−52.025	−5.548	−48.280	−7.009	−72.014
Gallic acid	−4.861	−24.020	−7.830	−72.009	−5.777	−37.897	−6.431	−54.483
Ampicillin	−5.733	−63.137	−9.041	−86.410	−6.299	−56.984	−8.234	−88.989

## Data Availability

The data presented in this study are available on request from the corresponding author.

## Supplementary Materials

The following supporting information can be downloaded at: https://www.mdpi.com/article/10.3390/plants14071071/s1.
