# Supplementary material for: Green Extraction Strategies and Bioactivity of Rheum cordatum Losinsk: Antioxidant, Antimicrobial, and Molecular Docking Insights
Source: Plants (Basel). 2025 Apr 1;14(7):1071. doi: 10.3390/plants14071071 (PMC11991350; doi:10.3390/plants14071071)
Supplement: Supplementary file 1 [file plants-14-01071-s001.zip › plants-3517900-supplementary.pdf]

Article

Green Extraction Strategies and Bioactivity of *Rheum cordatum* Losinsk: Antioxidant, Antimicrobial, and Molecular Docking Insights

Madina Amangeldinova [1,2], Mehmet Ersatır [3], Adem Necip [4], Mehmet Cimentepe [5], Nataliya Kudrina [1,2], Nina Terletskaya [1,2], Ozge Oztürk Cimentepe [6], Oguz Cakır [7], Mustafa Abdullah Yilmaz [8] and Metin Yildirim [9,*]

[1] Faculty of Biology and Biotechnology, Al-Farabi Kazakh National University, Al-Farabi Avenue 71, Almaty 050040, Kazakhstan; madu.ma@mail.ru (M.A.); kudrina_nat@mail.ru (N.K.); teni02@mail.ru (N.T.)
[2] Institute of Genetics and Physiology, Al-Farabi 93, Almaty 050040, Kazakhstan
[3] Department of Chemistry, Faculty of Art and Science, Cukurova Universiry, Adana 01330, Türkiye; mehmetersatir8@gmail.com
[4] Department of Pharmacy Services, Vocational School of Health Services, Harran University, Sanliurfa 63100, Türkiye; ademnecip@harran.edu.tr
[5] Department of Pharmaceutical Microbiology, Faculty of Pharmacy, Harran University, Sanliurfa 63100, Türkiye; mehmet.cimentepe@harran.edu.tr
[6] Department of Pharmacology, Faculty of Pharmacy, Harran University, Sanliurfa 63100, Türkiye; ozge.cimentepe@harran.edu.tr
[7] Department of Nutrition and Dietetics, Faculty of Health Sciences, Dicle University, Diyarbakir 21280, Türkiye; ocakir44@gmail.com
[8] Department of Analytical Chemistry, Faculty of Pharmacy, Dicle University, Diyarbakir 21280, Türkiye; mustafaabdullahyilmaz@gmail.com
[9] Department of Biochemistry, Faculty of Pharmacy, Harran University, Sanliurfa 63100, Türkiye
* Correspondence: metinyildirim4@gmail.com; Tel.: +90-5375274443

Supplementary Data	**Page Number**
LC-MS/MS based qualitative and quantitative identification of phytochemicals	S2
Mass spectrometer and chromatograph conditions	S2
**Fig. S1.** TIC (Total Ion Chromatogram) chromatogram of standard phenolic compounds	S3
**Fig. S2.** TIC (Total Ion Chromatogram) chromatogram of the extract obtained from the ultrasound-assisted extraction of *Rheum cordatum* Losinsk roots in ethanol for 1h	S3
**Fig. S3.** TIC (Total Ion Chromatogram) chromatogram of the extract obtained from the ultrasound-assisted extraction of *Rheum cordatum* Losinsk roots in ethanol for 4h	S4
**Fig. S4.** TIC (Total Ion Chromatogram) chromatogram of the extract obtained from the ultrasound-assisted extraction of *Rheum cordatum* Losinsk roots in methanol for 1h	S4
**Fig. S5.** TIC (Total Ion Chromatogram) chromatogram of the extract obtained from the ultrasound-assisted extraction of *Rheum cordatum* Losinsk roots in methanol for 4h	S5
**Fig. S6.** TIC (Total Ion Chromatogram) chromatogram of the extract obtained from *Rheum cordatum* Losinsk roots by supercritical CO_2 extraction at 100 atm pressure and 60°C temperature	S5
**Fig. S7.** TIC (Total Ion Chromatogram) chromatogram of the extract obtained from *Rheum cordatum* Losinsk roots by supercritical CO_2 extraction at 400 atm pressure and 60°C temperature	S6
**Fig. S8.** TIC (Total Ion Chromatogram) chromatogram of the extract obtained from *Rheum cordatum* Losinsk roots by subcritical ethanol extraction at 140 atm pressure and 60°C temperature	S6
**Fig. S9.** TIC (Total Ion Chromatogram) chromatogram of the extract obtained from *Rheum cordatum* Losinsk roots by subcritical ethanol extraction at 140 atm pressure and 80°C temperature	S7
**Table S1.** Analytical method validation parameters that belong to the LC-MS/MS method	S8

LC-MS/MS based qualitative and quantitative identification of phytochemicals

A previously developed and validated LC-MS/MS method was applied for the qualitative and quantitative evaluation of phytochemicals in *Rheum cordatum* Losinsk roots (Yilmaz, 2020) This method was used as the developed method is applicable not only to the selected species but also to a broad range of plant species. Using the recently developed LC/MS/MS technique, 53 phytochemical molecules, including 14 flavonoid aglycones, 13 flavonoid glycosides, 20 phenolic acids, three phenolic aldehydes, one benzopyrone, one stilbenoid glucoside, and one biflavonoid, were detected and quantified in the investigated species. Internal standard solutions were utilized to increase the reliability of the results by compensating for the matrix effects and analyte losses during the sample preparation and analyses. For flavonoid glycosides, flavonoids, and non-flavonoid substances, respectively, rutin D3, quercetin D3, and ferulic acid D3 were utilized as the deuterated internal standards. In-depth procedures of method validation have been previously described in literature in terms of inter-day and intra-day precision (repeatability), linearity, accuracy (recovery), detection and quantification limits (LOD/LOQ), and relative standard uncertainty (U% at 95% confidence level (k=2)). (Yilmaz, 2020)

Mass spectrometer and chromatograph conditions

A Shimadzu-Nexera model ultrahigh performance liquid chromatograph (UHPLC) coupled with a tandem mass spectrometer was used to accomplish quantiative evaluation of 53 phytochemicals. The reversed-phase UHPLC was equipped with an autosampler (SIL-30AC model), a column oven (CTO-10ASvp model), binary pumps (LC-30AD model), and a degasser (DGU- 20A3R model). The chromatographic seperation was performed on a reversed phase Agilent Poroshell 120 EC-C18 model (150 mm×2.1 mm, 2.7 µm) analytical column. The column temperature was set to 40°C. The elution gradient was composed of eluent A (water+5 mM ammonium formate+0.1% formic acid) and eluent B (methanol+5 mM ammonium formate+0.1% formic acid). The following gradient elution profile was used: 20-100% B (0-25 min), 100% B (25-35 min), 20% B (35-45 min). Furthermore, the solvent flow rate and injection volume were settled as 0.5 mL/min and 5 µL, respectively.

The mass spectrometric detection was carried out using a Shimadzu LCMS-8040 model tandem mass spectrometer equipped with an electrospray ionization (ESI) source operating in both negative and positive ionization modes. LC-ESI-MS/MS data were acquired and processed by LabSolutions software (Shimadzu). The MRM (multiple reaction monitoring) mode was used for the quantification of the phytochemicals. The MRM metot was optimized to selectively detect and quantify phytochemical compounds based on the screening of specified precursor phytochemical-to-fragment ion transitions. The collision energies (CE) were optimized in order to generate optimal phtochemical fragmentation and maximal transmission of the desired product ions. The MS operatinging conditions were applied as: drying gas (N2) flow, 15 L/min; nebulizing gas (N2) flow, 3 L/min; DL temperature, 250°C; heat block temperature, 400°C, and interface temperature, 350°C.

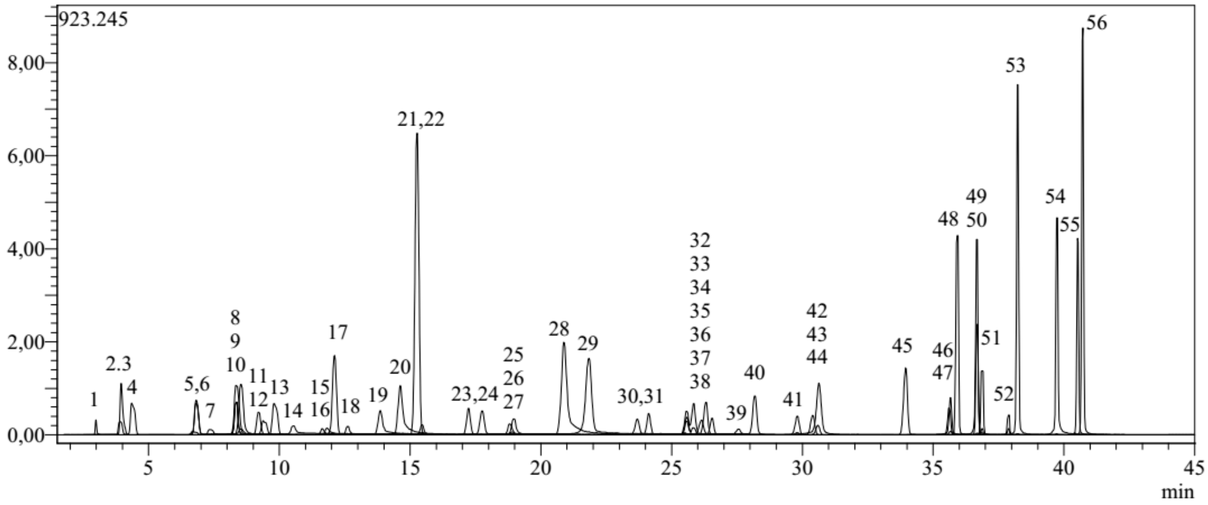

Fig. S1. TIC (Total Ion Chromatogram) chromatogram of standard phenolic compounds

(1:Quinic acid, 2: Fumaric acid, 3: Aconitic acid, 4: Gallic acid, 5: Epigallocatechin, 6: Protocatechuic acid, 7: Catechin, 8: Gentisic acid, 9: Chlorogenic acid, 10: Protocatechuic aldehyde, 11: Tannic acid, 12: Epigallocatechin gallate, 13: 1,5-dicaffeoylquinic acid, 14: 4-OH Benzoic acid, 15: Epicatechin, 16: Vanilic acid, 17: Caffeic acid, 18: Syringic acid, 19: Vanillin, 20: Syringic aldehyde, 21: Daidzin, 22: Epicatechin gallate, 23: Piceid, 24: p-Coumaric acid, 25: Ferulic acid D3, 26: Ferulic acid, 27: Sinapic acid, 28: Coumarin, 29: Salicylic acid, 30: Cynaroside, 31: Miquelianin, 32: Rutin, 33: Rutin D3, 34: isoquercitrin, 35: Hesperidin, 36: o-Coumaric acid, 37: Genistin, 38: Rosmarinic acid, 39: Ellagic acid, 40: Cosmosiin, 41: Quercitrin, 42: Astragalin, 43: Nicotiflorin, 44: Fisetin, 45: Daidzein, 46: Quercetin D3, 47: Quercetin, 48: Naringenin, 49: Hesperetin, 50: Luteolin, 51: Genistein, 52: Kaempferol, 53: Apigenin, 54: Amentoflavone, 55: Chrysin, 56: Acacetin) analysed by the developed LC–MS/MS method.

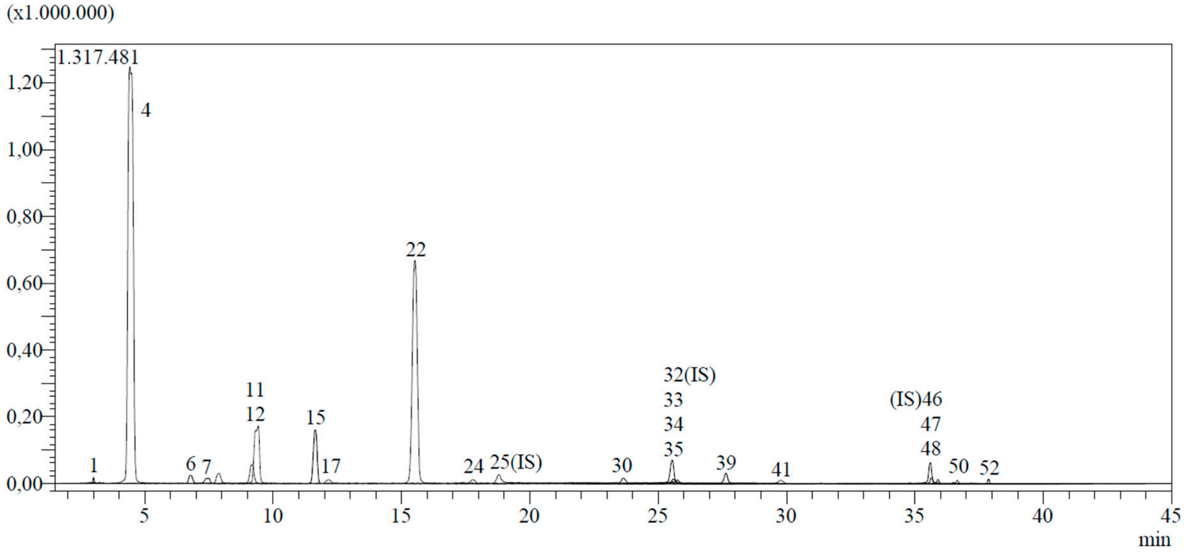

Fig. S2. TIC (Total Ion Chromatogram) chromatogram of the extract obtained from the ultrasound-assisted extraction of *Rheum cordatum* Losinsk roots in ethanol for 1h

1:Quinic acid, 4: Gallic acid, 6: Protocatechuic acid, 7: Catechin, 11: Tannic acid, 12: Epigallocatechin gallate, 15: Epicatechin, 17: Caffeic acid, 22: Epicatechin gallate, 24: p-Coumaric acid, 25: Ferulic acid D3, 30: Cynaroside, 32: Rutin, 33: Rutin D3, 34: isoquercitrin, 35: Hesperidin, 39: Ellagic acid, 41: Quercitrin, 46: Quercetin D3, 47: Quercetin, 48: Naringenin, 50: Luteolin, 52: Kaempferol

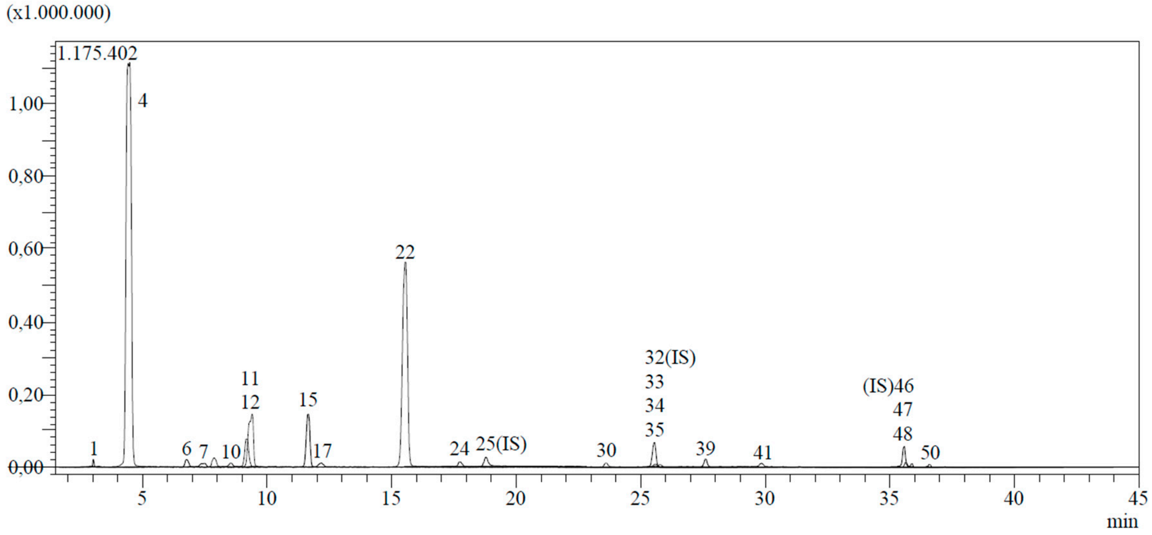

Fig. S3. TIC (Total Ion Chromatogram) chromatogram of the extract obtained from the ultrasound-assisted extraction of *Rheum cordatum* Losinsk roots in ethanol for 4h

1:Quinic acid, 4: Gallic acid, 6: Protocatechuic acid, 7: Catechin, 10: Protocatechuic aldehyde, 11: Tannic acid, 12: Epigallocatechin gallate, 15: Epicatechin, 17: Caffeic acid, 22: Epicatechin gallate, 24: p-Coumaric acid, 25: Ferulic acid D3, 30: Cynaroside, 32: Rutin, 33: Rutin D3, 34: isoquercitrin, 35: Hesperidin, 39: Ellagic acid, 41: Quercitrin, 46: Quercetin D3, 47: Quercetin, 48: Naringenin, 50: Luteolin

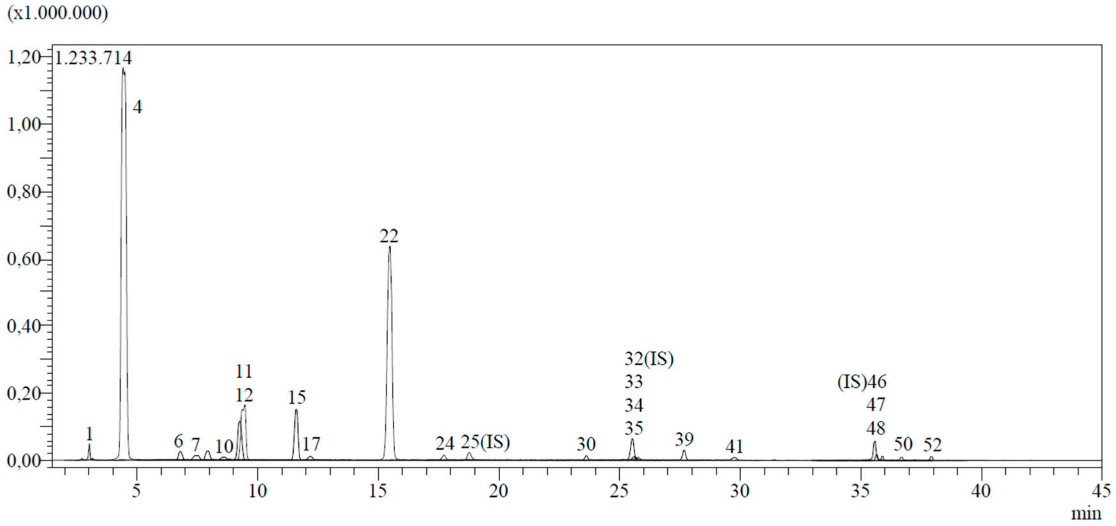

Fig. S4. TIC (Total Ion Chromatogram) chromatogram of the extract obtained from the ultrasound-assisted extraction of *Rheum cordatum* Losinsk roots in methanol for 1h

1:Quinic acid, 4: Gallic acid, 6: Protocatechuic acid, 7: Catechin, 10: Protocatechuic aldehyde, 11: Tannic acid, 12: Epigallocatechin gallate, 15: Epicatechin, 17: Caffeic acid, 22: Epicatechin gallate, 24: p-Coumaric acid, 25: Ferulic acid D3, 30: Cynaroside, 32: Rutin, 33: Rutin D3, 34: isoquercitrin, 35: Hesperidin, 39: Ellagic acid, 41: Quercitrin, 46: Quercetin D3, 47: Quercetin, 48: Naringenin, 50: Luteolin, 52: Kaempferol

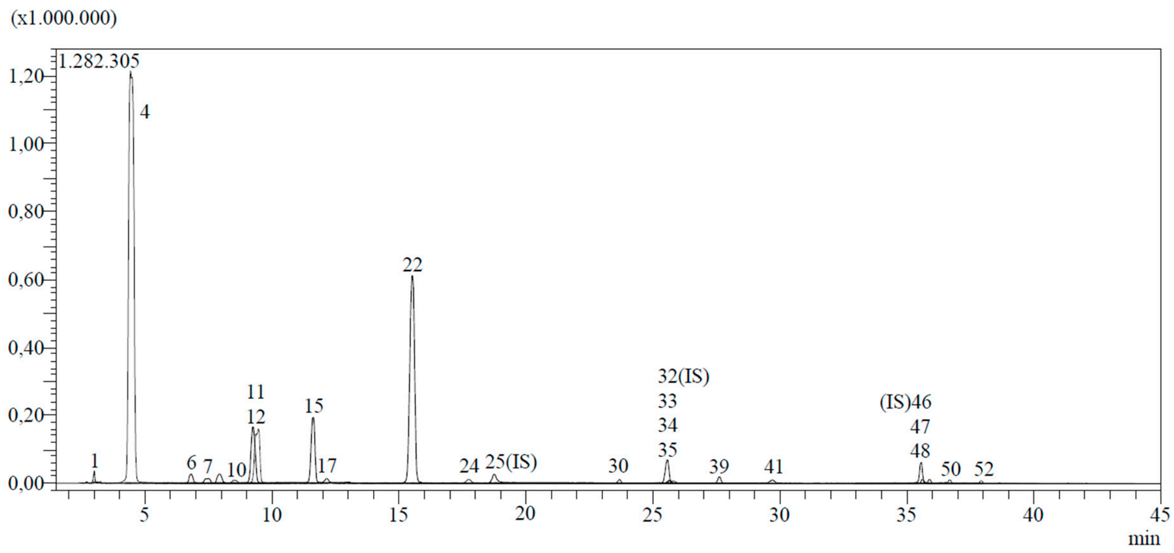

Fig. S5. TIC (Total Ion Chromatogram) chromatogram of the extract obtained from the ultrasound-assisted extraction of *Rheum cordatum* Losinsk roots in methanol for 4h

1:Quinic acid, Gallic acid, 6: Protocatechuic acid, 7: Catechin, 10: Protocatechuic aldehyde, 11: Tannic acid, 12: Epigallocatechin gallate, 15: Epicatechin, 17: Caffeic acid, 22: Epicatechin gallate, 24: p-Coumaric acid, 25: Ferulic acid D3, 30: Cynaroside, 32: Rutin, 33: Rutin D3, 34: isoquercitrin, 35: Hesperidin, 39: Ellagic acid, 41: Quercitrin, 46: Quercetin D3, 47: Quercetin, 48: Naringenin, 50: Luteolin, 52: Kaempferol

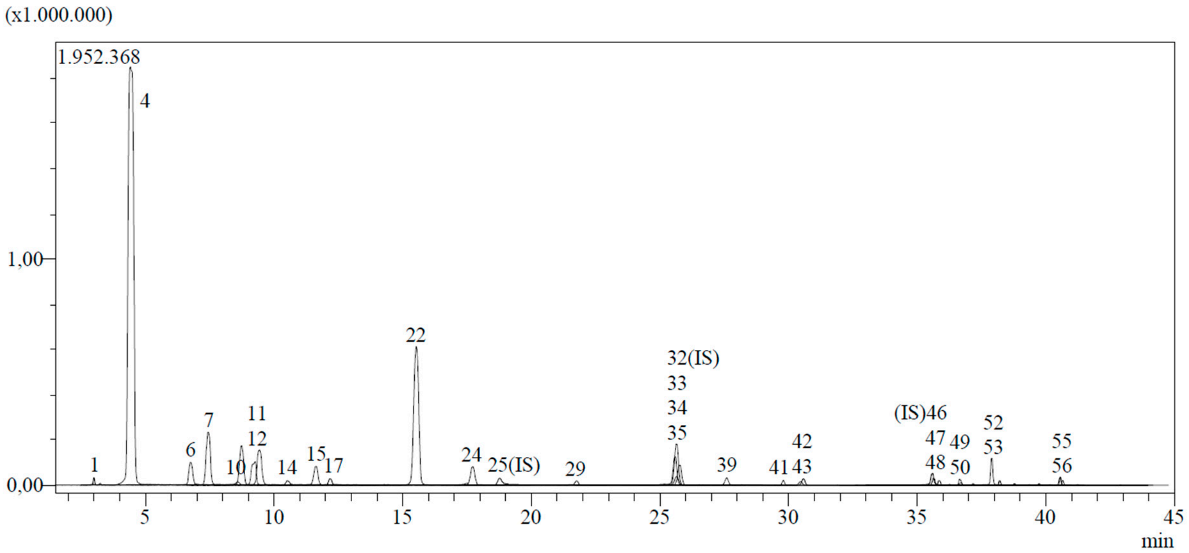

Fig. S6. TIC (Total Ion Chromatogram) chromatogram of the extract obtained from *Rheum cordatum* Losinsk roots by supercritical CO_2 extraction at 100 atm pressure and 60°C temperature

1:Quinic acid, 4: Gallic acid, 6: Protocatechuic acid, 7: Catechin, 10: Protocatechuic aldehyde, 11: Tannic acid, 12: Epigallocatechin gallate, 14: 4-OH Benzoic acid, 15: Epicatechin, 17: Caffeic acid, 22: Epicatechin gallate, 24: p-Coumaric acid, 25: Ferulic acid D3, 29: Salicylic acid, 32: Rutin, 33: Rutin D3, 34: isoquercitrin, 35: Hesperidin, 39: Ellagic acid, 41: Quercitrin, 42: Astragalin, 43: Nicotiflorin, 46: Quercetin D3, 47: Quercetin, 48: Naringenin, 49: Hesperetin, 50: Luteolin, 52: Kaempferol, 53: Apigenin, 55: Chrysin, 56: Acacetin

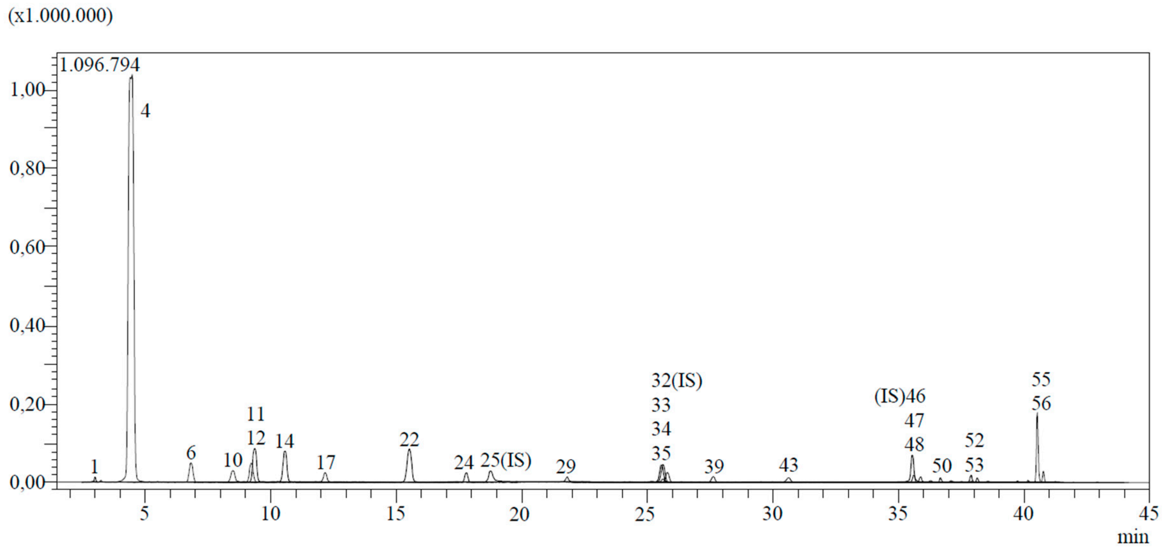

Fig. S7. TIC (Total Ion Chromatogram) chromatogram of the extract obtained from *Rheum cordatum* Losinsk roots by supercritical CO_2 extraction at 400 atm pressure and 60°C temperature

1:Quinic acid, 4: Gallic acid, 6: Protocatechuic acid, 10: Protocatechuic aldehyde, 11: Tannic acid, 12: Epigallocatechin gallate, 14: 4-OH Benzoic acid, 17: Caffeic acid, 22: Epicatechin gallate, 24: p-Coumaric acid, 25: Ferulic acid D3, 29: Salicylic acid, 32: Rutin, 33: Rutin D3, 34: isoquercitrin, 35: Hesperidin, 39: Ellagic acid, 43: Nicotiflorin, 46: Quercetin D3, 47: Quercetin, 48: Naringenin, 50: Luteolin, 52: Kaempferol, 53: Apigenin, 55: Chrysin, 56: Acacetin

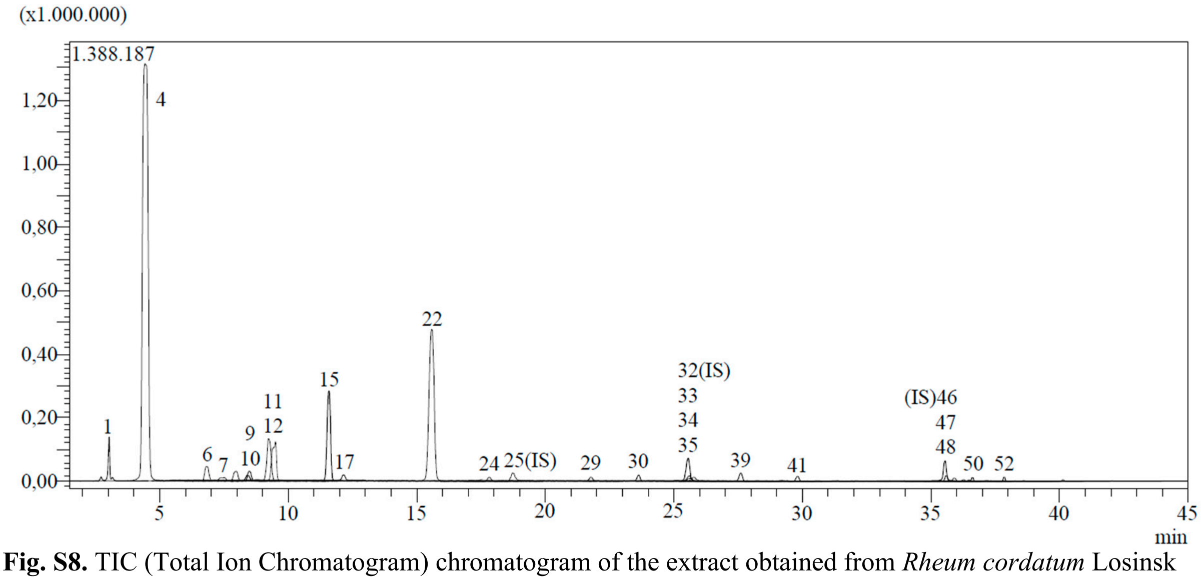

Fig. S8. TIC (Total Ion Chromatogram) chromatogram of the extract obtained from *Rheum cordatum* Losinsk roots by subcritical ethanol extraction at 140 atm pressure and 60°C temperature

1:Quinic acid, 4: Gallic acid, 6: Protocatechuic acid, 7: Catechin, 9: Chlorogenic acid, 10: Protocatechuic aldehyde, 11: Tannic acid, 12: Epigallocatechin gallate, 15: Epicatechin, 17: Caffeic acid, 22: Epicatechin gallate, 24: p-Coumaric acid, 25: Ferulic acid D3, 29: Salicylic acid, 30: Cynaroside, 32: Rutin, 33: Rutin D3, 34: isoquercitrin, 35: Hesperidin, 39: Ellagic acid, 41: Quercitrin, 46: Quercetin D3, 47: Quercetin, 48: Naringenin, 50: Luteolin, 52: Kaempferol

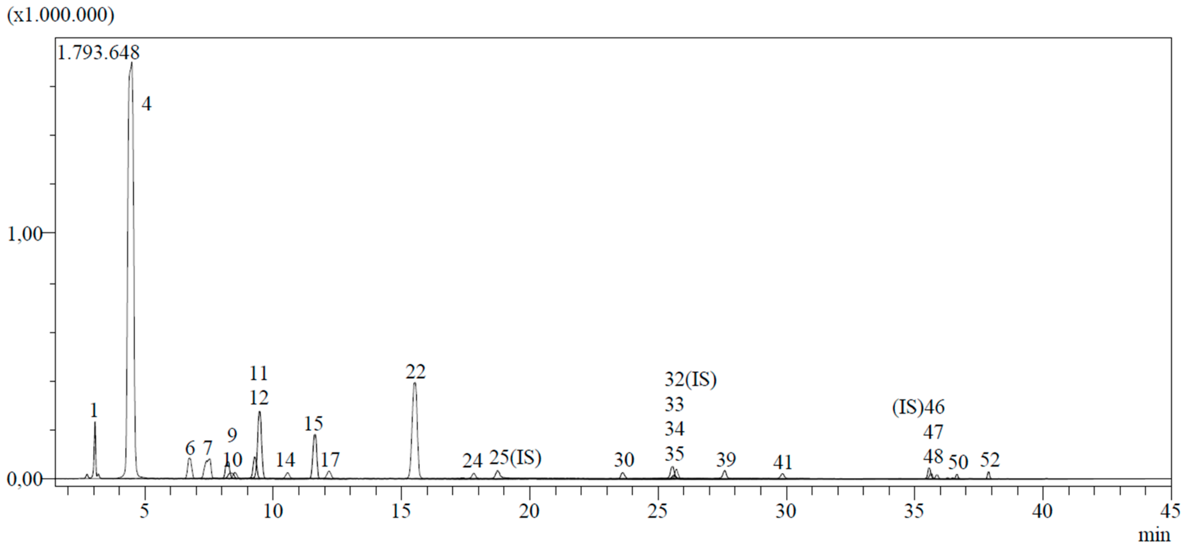

Fig. S9. TIC (Total Ion Chromatogram) chromatogram of the extract obtained from *Rheum cordatum* Losinsk roots by subcritical ethanol extraction at 140 atm pressure and 80°C temperature

1:Quinic acid, 4: Gallic acid, 6: Protocatechuic acid, 7: Catechin, 9: Chlorogenic acid, 10: Protocatechuic aldehyde, 11: Tannic acid, 12: Epigallocatechin gallate, 14: 4-OH Benzoic acid, 15: Epicatechin, 17: Caffeic acid, 22: Epicatechin gallate, 24: p-Coumaric acid, 25: Ferulic acid D3, 30: Cynaroside, 32: Rutin, 33: Rutin D3, 34: isoquercitrin, 35: Hesperidin, 39: Ellagic acid, 41: Quercitrin, 46: Quercetin D3, 47: Quercetin, 48: Naringenin, 50: Luteolin, 52: Kaempferol

Table S1. Analytical method validation parameters that belong to the LC-MS/MS method

No	Analytes	RT[a]	M.I. (m/z)[b]	F.I. (m/z)[c]	Ion. mode	Equation	r^2[d]	RSD%[e] Interday	RSD%[e] Intraday	Linearity Range (mg/L)	LOD/LOQ (µg/L)[f]	Recovery (%) Interday	Recovery (%) Intraday	U[g]	Gr. No[i]
1	Quinic acid	3.0	190.8	93.0	Neg	*y*=-0.0129989+2.97989×	0.996	0.69	0.51	0.1-5	25.7/33.3	1.0011	1.0083	0.0372	1
2	Fumaric aid	3.9	115.2	40.9	Neg	*y*=-0.0817862+1.03467×	0.995	1.05	1.02	1-50	135.7/167.9	0.9963	1.0016	0.0091	1
3	Aconitic acid	4.0	172.8	129.0	Neg	*y*=-0.7014530+32.9994×	0.971	2.07	0.93	0.1-5	16.4/31.4	0.9968	1.0068	0.0247	1
4	Gallic acid	4.4	168.8	79.0	Neg	*y*=0.0547697+20.8152×	0.999	1.60	0.81	0.1-5	13.2/17.0	1.0010	0.9947	0.0112	1
5	Epigallocatechin	6.7	304.8	219.0	Neg	*y*=-0.00494986+0.0483704×	0.998	1.22	0.73	1-50	237.5/265.9	0.9969	1.0040	0.0184	3
6	Protocatechuic acid	6.8	152.8	108.0	Neg	*y*=0.211373+12.8622×	0.957	1.43	0.76	0.1-5	21.9/38.6	0.9972	1.0055	0.0350	1
7	Catechin	7.4	288.8	203.1	Neg	*y*=-0.00370053+0.431369×	0.999	2.14	1.08	0.2-10	55.0/78.0	1.0024	1.0045	0.0221	3
8	Gentisic acid	8.3	152.8	109.0	Neg	*y*=-0.0238983+12.1494×	0.997	1.81	1.22	0.1-5	18.5/28.2	0.9963	1.0077	0.0167	1
9	Chlorogenic acid	8.4	353.0	85.0	Neg	*y*=0.289983+36.3926×	0.995	2.15	1.52	0.1-5	13.1/17.6	1.0000	1.0023	0.0213	1
10	Protocatechuic aldehyde	8.5	137.2	92.0	Neg	*y*=0.257085+25.4657×	0.996	2.08	0.57	0.1-5	15.4/22.2	1.0002	0.9988	0.0396	1
11	Tannic acid	9.2	182.8	78.0	Neg	*y*=0.0126307+26.9263×	0.999	2.40	1.16	0.05-2.5	15.3/22.7	0.9970	0.9950	0.0190	1
12	Epigallocatechin gallate	9.4	457.0	305.1	Neg	*y*=-0.0380744+1.61233×	0.999	1.30	0.63	0.2-10	61.0/86.0	0.9981	1.0079	0.0147	3
13	1,5-dicaffeoylquinic acid	9.8	515.0	191.0	Neg	*y*=-0.0164044+16.6535×	0.999	2.42	1.48	0.1-5	5.8/9.4	0.9983	0.9997	0.0306	1
14	4-OH Benzoic acid	10.5	137,2	65.0	Neg	*y*=-0.0240747+5.06492×	0.999	1.24	0.97	0.2-10	68.4/88.1	1.0032	1.0068	0.0237	1
15	Epicatechin	11.6	289.0	203.0	Neg	*y*=-0.0172078+0.0833424×	0.996	1.47	0.62	1-50	139.6/161.6	1.0013	1.0012	0.0221	3
16	Vanilic acid	11.8	166.8	108.0	Neg	*y*=-0.0480183+0.779564×	0.999	1.92	0.76	1-50	141.9/164.9	1.0022	0.9998	0.0145	1
17	Caffeic acid	12.1	179.0	134.0	Neg	*y*=0.120319+95.4610×	0.999	1.11	1.25	0.05-2.5	7.7/9.5	1.0015	1.0042	0.0152	1
18	Syringic acid	12.6	196.8	166.9	Neg	*y*=-0.0458599+0.663948×	0.998	1.18	1.09	1-50	82.3/104.5	1.0006	1.0072	0.0129	1
19	Vanillin	13.9	153.1	125.0	Poz	*y*=0.00185898+20.7382×	0.996	1.10	0.85	0.1-5	24.5/30.4	1.0009	0.9967	0.0122	1
20	Syringic aldehyde	14.6	181.0	151.1	Neg	*y*=-0.0128684+7.90153×	0.999	2.51	0.77	0.4-20	19.7/28.0	1.0001	0.9964	0.0215	1
21	Daidzin	15.2	417.1	199.0	Poz	*y*=9.45747+152.338×	0.996	2.25	1.32	0.05-2.5	7.0/9.5	0.9955	1.0017	0.0202	2
22	Epicatechin gallate	15.5	441.0	289.0	Neg	*y*=-0.0142216+1.06768×	0.997	1.63	1.28	0.1-5	19.5/28.5	0.9984	0.9946	0.0229	3
23	Piceid	17.2	391.0	135/106.9	Poz	*y*=0.00772525+25.4181×	0.999	1.94	1.16	0.05-2.5	13.8/17.8	1.0042	0.9979	0.0199	1
24	*p*-Coumaric acid	17.8	163.0	93.0	Neg	*y*=0.0249034+18.5180×	0.999	1.92	1.43	0.1-5	25.9/34.9	1.0049	1.0001	0.0194	1
25	Ferulic acid-D3-IS[h]	18.8	196.2	152.1	Neg	N.A.	N.A.	N.A.	N.A.	N.A.	N.A.	N.A.	N.A.	0.0170	1
26	Ferulic acid	18.8	192.8	149.0	Neg	*y*=-0.0735254+1.34476×	0.999	1.44	0.53	1-50	11.8/15.6	0.9951	0.9976	0.0181	1
27	Sinapic acid	18.9	222.8	193.0	Neg	*y*=-0.0929932+0.836324×	0.999	1.45	0.52	0.2-10	65.2/82.3	1.0031	1.0037	0.0317	1
28	Coumarin	20.9	146.9	103.1	Poz	*y*=0.0633397+136.508×	0.999	2.11	1.54	0.05-2.5	214.2/247.3	0.9950	0.9958	0.0383	1

[a]R.T.: Retention time, [b]MI *(m/z):* Molecular ions of the standard analytes (m/z ratio), [c]FI *(m/z):* Fragment ions [d]r^2: Coefficient of determination, [e]*RSD*: Relative standard deviation, [f]*LOD*/*LOQ* (µg/L): Limit of detection/quantification, [g]*U* (%): percent relative uncertainty at 95% confidence level ($k = 2$), [h]IS: Internal standard, [i]Gr. No: Represents grouping of internal standards, these numbers indicate which IS stands for which phenolic compound.

Table 2. Analytical method validation parameters that belong to the LC-MS/MS method (Continued)

No	Analytes	RT[a]	M.I. (m/z)[b]	F.I. (m/z)[c]	Ion. mode	Equation	r^2[d]	*RSD*%[e]		Linearity Range (mg/L)	*LOD*/*LOQ* (µg/L)[f]	Recovery (%)		*U*[g]	Gr. No
								Interday	Intraday			Interday	Intraday		
29	Salicylic acid	21.8	137.2	65.0	Neg	*y*=0.239287+153.659×	0.999	1.48	1.18	0.05-2.5	6.0/8.3	0.9950	0.9998	0.0158	1
30	Cynaroside	23.7	447.0	284.0	Neg	*y*=0.280246+6.13360×	0.997	1.56	1.12	0.05-2.5	12.1/16.0	1.0072	1.0002	0.0366	2
31	Miquelianin	24.1	477.0	150.9	Neg	*y*=-0.00991585+5.50334×	0.999	1.31	0.95	0.1-5	10.6/14.7	0.9934	0.9965	0.0220	2
32	Rutin-D3-IS[h]	25.5	612.2	304.1	Neg	N.A.	N.A.	N.A.	N.A.	N.A.	N.A.	N.A.	N.A.	N.A.	2
33	Rutin	25.6	608.9	301.0	Neg	*y*=-0.0771907+2.89868×	0.999	1.38	1.09	0.1-5	15.7/22.7	0.9977	1.0033	0.0247	2
34	isoquercitrin	25.6	463.0	271.0	Neg	*y*=-0.111120+4.10546×	0.998	2.13	0.78	0.1-5	8.7/13.5	1.0057	0.9963	0.0220	2
35	Hesperidin	25.8	611.2	449.0	Poz	*y*=0.139055+13.2785×	0.999	1.84	1.35	0.1-5	19.0/26.0	0.9967	1.0043	0.0335	2
36	*o*-Coumaric acid	26.1	162.8	93.0	Neg	*y*=0.00837193+11.2147×	0.999	2.11	1.46	0.1-5	31.8/40.4	1.0044	0.9986	0.0147	1
37	Genistin	26.3	431.0	239.0	Neg	*y*=1.65808+7.57459×	0.991	2.01	1.28	0.1-5	14.9/21.7	1.0062	1.0047	0.0083	2
38	Rosmarinic acid	26.6	359.0	197.0	Neg	*y*=-0.0117238+8.04377×	0.999	1.24	0.86	0.1-5	16.2/21.2	1.0056	1.0002	0.0130	1
39	Ellagic acid	27.6	301.0	284.0	Neg	*y*=0.00877034+0.663741×	0.999	1.57	1.23	0.4-20	56.9/71.0	1.0005	1.0048	0.0364	1
40	Cosmosiin	28.2	431.0	269.0	Neg	*y*=-0.708662+8.62498×	0.998	1.65	1.30	0.1-5	6.3/9.2	0.9940	0.9973	0.0083	2
41	Quercitrin	29.8	447.0	301.0	Neg	*y*=-0.00153274+3.20368×	0.999	2.24	1.16	0.1-5	4.8/6.4	0.9960	0.9978	0.0268	2
42	Astragalin	30.4	447.0	255.0	Neg	*y*=0.00825333+3.51189×	0.999	2.08	1.72	0.1-5	6.6/8.2	0.9968	0.9957	0.0114	2
43	Nicotiflorin	30.6	592.9	255.0/284.0	Neg	*y*=0.00499333+2.62351×	0.999	1.48	1.23	0.05-2.5	11.9/16.7	0.9954	1.0044	0.0108	2
44	Fisetin	30.6	285.0	163.0	Neg	*y*=0.0365705+8.09472×	0.999	1.75	1.19	0.1-5	10.1/12.7	0.9980	1.0042	0.0231	3
45	Daidzein	34.0	253.0	223.0	Neg	*y*=-0.0329252+6.23004×	0.999	2.18	1.73	0.1-5	9.8/11.6	0.9926	0.9963	0.0370	3
46	Quercetin-D3-IS[h]	35.6	304.0	275.9	Neg	N.A.	N.A.	N.A.	N.A.	N.A.	N.A.	N.A.	N.A.	N.A.	3
47	Quercetin	35.7	301.0	272.9	Neg	*y*=+0.00597342+3.39417×	0.999	1.89	1.38	0.1-5	15.5/19.0	0.9967	0.9971	0.0175	3
48	Naringenin	35.9	270.9	119.0	Neg	*y*=-0.00393403+14.6424×	0.999	2.34	1.69	0.1-5	2.6/3.9	1.0062	1.0020	0.0392	3
49	Hesperetin	36.7	301.0	136.0/286.0	Neg	*y*=+0.0442350+6.07160×	0.999	2.47	2.13	0.1-5	7.1/9.1	0.9998	0.9963	0.0321	3
50	Luteolin	36.7	284.8	151.0/175.0	Neg	*y*=-0.0541723+30.7422×	0.999	1.67	1.28	0.05-2.5	2.6/4.1	0.9952	1.0029	0.0313	3
51	Genistein	36.9	269.0	135.0	Neg	*y*=-0.00507501+12.1933×	0.999	1.48	1.19	0.05-2.5	3.7/5.3	1.0069	1.0012	0.0337	3
52	Kaempferol	37.9	285.0	239.0	Neg	*y*=-0.00459557+3.13754×	0.999	1.49	1.26	0.05-2.5	10.2/15.4	0.9992	0.9990	0.0212	3
53	Apigenin	38.2	268.8	151.0/149.0	Neg	*y*=0.119018+34.8730×	0.998	1.17	0.96	0.05-2.5	1.3/2.0	0.9985	1.0003	0.0178	3
54	Amentoflavone	39.7	537.0	417.0	Neg	*y*=0.727280+33.3658×	0.992	1.35	1.12	0.05-2.5	2.8/5.1	0.9991	1.0044	0.0340	3
55	Chrysin	40.5	252.8	145.0/119.0	Neg	*y*=-0.0777300+18.8873×	0.999	1.46	1.21	0.05-2.5	1.5/2.8	0.9922	1.0050	0.0323	3
56	Acacetin	40.7	283.0	239.0	Neg	*y*=-0.559818+163.062×	0.997	1.67	1.28	0.02-1	1.5/2.5	0.9949	1.0011	0.0363	3

[a]R.T.: Retention time, [b]MI (*m/z)*: Molecular ions of the standard analytes (m/z ratio), [c]FI (*m/z)*: Fragment ions [d]r^2: Coefficient of determination, [e]*RSD*: Relative standard deviation, [f]*LOD*/*LOQ* (µg/L): Limit of detection/quantification, [g]*U* (%): percent relative uncertainty at 95% confidence level (k = 2), [h]IS: Internal standard, [i]Gr. No: Represents grouping of internal standards, these numbers indicate which IS stands for which phenolic compound.

References

Yilmaz, M.A., 2020. Simultaneous quantitative screening of 53 phytochemicals in 33 species of medicinal and aromatic plants: A detailed, robust and comprehensive LC–MS/MS method validation. Industrial Crops and Products, 149, p.112347. https://doi.org/10.1016/j.indcrop.2020.112347
